# Development of the Ileal Microbiota in Three Broiler Breeds

**DOI:** 10.3389/fvets.2020.00017

**Published:** 2020-01-30

**Authors:** Peter Richards-Rios, Jo Fothergill, Marion Bernardeau, Paul Wigley

**Affiliations:** ^1^Department of Health and Life Sciences, Institute of Infection and Global Health, University of Liverpool, Liverpool, United Kingdom; ^2^DuPont Industrial Biosciences, Genencor International BV, Leiden, Netherlands

**Keywords:** microbiota, chicks, broilers, illumina, microbiology

## Abstract

The development and succession of the microbiota in ileal mucus and lumen samples from three breeds of broiler chicken (Cobb 500, *n* = 36; Hubbard JA87, *n* = 38; and Ross 308, *n* = 36) was observed between 3 and 42 days post hatch (d.p.h). Chicks were housed in the same room of a climate-controlled, biosecure chicken housing unit. Between 0 and 14 d.p.h, chicks were kept in three circular brooder pens ensuring a mixture of breeds in each brooder. From 22 d.p.h, chicks were removed from the brooders and kept in the same room. DNA was extracted from a pooled sample of ileal mucus and luminal contents taken from five birds of each breed at 3, 7, 14, 21, 28, and 42 d.p.h. High-throughput Illumina sequencing was performed for the V4 hypervariable region of the 16S rRNA gene. The initial microbiota in the ileum varied between breeds. The common features were a low diversity and general dominance by one or two taxa such as *Enterococcus* or *Escherichia* with relatively low numbers of *Lactobacillus*. *Escherichia* became the most abundant genus in samples where *Enterococcus* was previously the dominant taxa. The next phase of development was marked by an increase in the abundance of Candidatus *Arthromitus* in the mucus and *Lactobacillus* in the lumen. The high abundance of Candidatus *Arthromitus* persisted between 7 and 14 d.p.h after which *Lactobacillus* became the most abundant genus in both the mucus and lumen. Dominance of the ileal microbiota by *Lactobacillus* was a transient feature. By 42 d.p.h, the relative abundance of *Lactobacillus* had fallen while a range of other taxa including *Escherichia, Turicibacter*, and members of Clostridiales increased. This general pattern was followed by all breeds, however, the rate at which succession occurred differed as Ross matured quicker than Cobb with Hubbard as an intermediate.

## 1. Introduction

The intestinal microbiota of an individual chicken may be composed of between 200 and 350 different bacterial species (1) while around 640 bacterial species have so far been identified in the chicken gastrointestinal tract (2). In recent years it has become apparent that this diverse range of bacteria are not innocuous bystanders but play a range of roles in the host, from metabolism to immune maturation (3). Advances in the field have spurred efforts to identify beneficial bacteria and modulate their abundance to accentuate their effects. In the face of antibiotic resistance and a need to find new approaches to infectious disease control, the question of how certain bacteria modulate host immunity is of particular interest to the broiler industry which produces chickens for meat. While some bacteria have been identified as potential candidates for immunomodulatory probiotics there are still gaps in our knowledge regarding the normal development of the ileal microbiota in chickens. Recent observational studies of the ileal microbiota are limited. Most of the initial studies were conducted using techniques such as denaturing gradient gel electrophoresis and clone libraries which have been superseded by next generation sequencing (NGS) technology. More recent studies which have used NGS focus on differences in microbiota composition between a treatment group and an untreated control. This study aims to revisit the topic of normal ileal microbiota development using the increased resolution of NGS to shed light on microbial succession.

The small intestine is often divided into three anatomical sections, the duodenum, jejunum, and ileum. The duodenum is often defined as the first loop of the small intestine adjacent to the pancreas with the jejunum extending until Meckel's diverticulum and the ileum from there to the ileo-caeco-colic junction (4). As in mammals, digesta is mixed with digestive enzymes and bile in the duodenum where fat digestion takes place (5). However, digesta is not detained for long in the duodenum with a relatively short retention time of ~5 min (6). The majority of digestion and absorption occurs in the duodenum (4). Despite its essential role in digestion and absorption, digesta is only retained in the jejunum for 40 to 60 min (7, 8). The function of the ileum is largely water and mineral absorption, however, there is evidence that the ileum plays a role in starch and fat digestion in fast-growing broiler chickens (9–11). In terms of amino acid metabolism, although the ileum does not play a role in absorption the ileal microbiota plays an important role as an amino acid sink. Even with an optimal diet some protein will escape host absorption and enter the distal gastrointestinal system. Free dietary amino acids in the caecum are liable to be fermented by commensals, undergoing putrefaction which produces toxic end-products (12). In this respect, a healthy population of amino acid absorbing bacteria in the ileum would be beneficial.

Despite their differences in digestive function, there appears to be little difference in microbiome composition between the three small intestinal compartments (13), although the density of the microbiota is lower in the proximal small intestine compared to the ileum (12). This allows the inclusion of results from studies where samples from small intestinal compartments were pooled or the authors did not specify which section was sampled when discussing current literature regarding the ileal microbiota. As in the crop and gizzard, the small intestine has been found to be dominated by *Lactobacillus* with several studies identifying between 70 and 90% of sequences as belonging to this genus (13–17). However, the most abundant species of *Lactobacillus* often differs between the crop and small intestine. For example, Witzig et al. (17) found that *L. salivarius* was the most abundant species in the crop with a relative abundance of > 46% while *L. crispatus* was the most abundant in the jejunum (> 81%) and ileum > 77%. Early studies of the small intestinal microbiome suggested that other taxa were also present including Enterococcaceae, Streptococcaceae, and Clostridiaceae (15, 16). With the advent of 16S rRNA gene sequencing and more complete databases for taxonomic assignment of taxa, a greater diversity of taxa have been identified in the small intestine. These have included Peptostreptococcaceae, Turicibacteraceae, Bifidobacteriaceae, and Erysipelotrichaceae (18, 19). Candidatus *Athromitus* is also increasingly identified within the early ileal microbiome and has been associated with increased performance although later colonization with this taxa is associated with poor performance (20). There is also evidence to suggest that the mucus associated microbiome is different to the luminal microbiome with a lower relative abundance of Lactobacillaceae and a more diverse microbiota including Peptostreptococcaceae, Lachnospiraceae, Burkholderiaceae, and Ruminococcaceae (16, 21).

The pattern of succession that results in the adult ileal microbiota is less well-characterized. The microbiota of day-old chicks has previously been investigated, although care should be taken when interpreting results as chicks are classified by hatcheries as “day-old” up to 72 h post-hatch. Studies examining the microbiota of day old chicks tend to focus on the caecum, however, since the caecal and ileal microbiota are similar until around 3 days post hatch (d.p.h), the ileal microbiota can be inferred from that of the caecum (16, 22). Microbes inhabiting the gut immediately after hatch are derived from the environment during incubation, hatching and handling during delivery. This initial colonization is dependent upon the presence of environmental bacteria and has been found to differ significantly between hatcheries (23). This likely explains the wide variety of results obtained by different groups when examining the intestinal microbiota in day-old chicks. In general, the initial microbiome is dominated Enterobacteriaceae or Clostridiaceae, although there are reports of high abundance other taxa such as Streptococcaceae and Enterococcaceae (3, 20, 24, 25). At the genus level, the most commonly isolated Enterobacteriaceae are *Escherichia/Shigella* while Clostridiaceae are most often assigned to *Clostridium sensu stricto* 1. Both of these genera are known to contain potential pathogens such as *Clostridium perfringens*. These bacteria are likely to be environmental in origin, deriving either from hatchery equipment or workers. Although the possibility of maternal microbiota transfer via the reproductive tract has been claimed the most abundant taxa in embryonic gut and egg albumin was *Psuedomonas* with no presence of Enterobacteriaceae of Clostridiaceae (26).

As previously mentioned, the ileal microbiome remains similar to the caecal microbiome during the first few days of life. Wise and Siragusa (27) examined the bacterial community in the ileum and discovered that Enterobacteriaceae followed a trend of decline as in the caecum. This decline was associated with increased abundance of *Lactobacillus* which replaced Enterobacteriaceae as the dominant taxa by 14 d.p.h (27). Schokker et al. (28) described a faster transition to higher abundance of *Lactobacillus*. The day-old microbiome was dominated by Enterococcaceae with a high proportion of *Escherichia* sequences and *Lactobacillus* present but in very low numbers. By 4 d.p.h, the balance had reversed with *Lactobacillus* accounting for up to 88% of sequences associated with a decline in Enterococcaceae and *Escherichia*. Aside from *Lactobacillus*, there was an increase in microbial diversity with age with *Streptococcus* and some Clostridia contributing small numbers of sequences (28). The succession of *Lactobacillus* species in the ileum has not been studied in detail. Johnson et al. (20) reported seven species of *Lactobacillus* in the ileum of chickens between 0 and 42 d.p.h. Only two of these species, *L. salivarius* and *L. crispatus*, were present at 0 d.p.h, with four at 7 d.p.h and all seven at 21 d.p.h. Candidatus *Athromitus* colonizes the ileum between 7 and 14 d.p.h (20, 25) and has been reported as the prominent taxa in the ileal mucus although *Lactobacillus* reclaims dominance at later time points (29). Other slow growing taxa such as *Romboutsia*, a member of Peptostreptococcaceae, begin to colonize the ileum from 10 to 21 d.p.h.

This study aims to revisit the topic of normal ileal microbiota development in the lumen and mucus using the increased resolution of next generation sequencing to shed light on microbial succession. The second objective of this study was to observe the development of the ileal microbiota in three common breeds of broiler chicken (Cobb 500, Hubbard JA87, and Ross 308) whilst they are housed together.

## 2. Materials and Methods

### 2.1. Animals and Housing

One hundred and ten (36 Cobb 500, 38 Hubbard JA87, and 36 Ross 308) “day-old” chicks were obtained from a single commercial hatchery. Chicks were distributed across three circular brooder pens (2 m diameter) in the same room of a climate-controlled, biosecure chicken housing unit. Each brooder used a wood shaving substrate and contained the same number of chicks from each breed. Chicks were tagged with colored wing tags to allow accurate identification of the different breeds. Water and feed were provided *ad libitum* by a drinker and feeder in each brooder. Chicks were fed a pelleted vegetable protein-based starter diet (Special Diet Services, Witham, Essex, UK) until 14 days post hatch (d.p.h). From 14 d.p.h a pelleted vegetable protein-based grower diet (Special Diet Services, Witham, Essex, UK) was provided until the end of the experiment. Nutritional composition of the starter and grower diets is displayed in Table 1 with a full list of ingredients and additives provided in the Supplementary Materials. No coccidiostats or antimicrobials were added to either diet due to the high biosecurity levels maintained in the housing. At 22 d.p.h the birds no longer required brooder lamps and, as such, they were removed from the brooders and housed together in the same room on wood shavings. Temperature in the birds' pens was maintained between 25 and 30°C. No mortality was observed during the study. All experimental protocols were conducted in accordance with the Animals (Scientific Procedures) Act 1986 under project license 40/3652 and was approved by the University of Liverpool Animal Welfare and Ethical Review Body prior to the award of the license.

**Table 1:** Composition of starter and grower diets.

**Analytical constituents (%)**	**Diet**	
	**Starter**	**Grower**
Crude fat	2.7	2.4
Crude protein	18.9	15.6
Crude fiber	3.8	4.1
Crude ash	6.6	5.6
Lysine	0.99	0.69
Methionine	0.44	0.27
Calcium	1.05	0.89
Phosphorus	0.7	0.62
Sodium	0.15	0.15
Magnesium	0.17	0.22
Copper	15 mg/kg	16 mg/kg

### 2.2. Sample Collection

Five chickens of each breed were euthanized for sample collection at 3, 7, 14, 21, 28, and 42 d.p.h giving a total of 15 birds sampled at each time point. After euthanasia by cervical dislocation the abdomen was sprayed with 70% ethanol. Skin incisions were made to expose the sternum which was then reflected to give good access to the coelom. The ileum, identified as the section of small intestine between Meckel's diverticulum and the ileo-caeco-colic junction, was removed. Approximately 5 cm of ileum was cut from the middle of the organ and the contents manually expressed into a sterile container. Any visible digesta remaining was manually expressed but the ileal section was not rinsed before sampling the mucus layer. The ileal section was opened longitudinally using a sterile scalpel which was then used to gently scrape off the mucus layer and transfer the mucus to a sterile container. Mucus samples were taken from 14 d.p.h as the ileum at earlier time points was too small to yield adequate mucus for accurate pooling. Samples from 3 d.p.h were weighed and pooled by breed as some chicks yielded less than 200 mg of content. For all other time points, 200 mg of ileal content from each bird was taken and pooled by breed. Mucus samples were weighed, diluted with 500 μ l of sterile water, pooled by breed and homogenized. The pooled samples were flash frozen in liquid nitrogen and stored at –20°C for 5 weeks before DNA extraction. Samples from the caecum were also collected with the results published separately (30).

### 2.3. DNA Extraction

DNA was extracted from each sample using Zymobiomics DNA MiniKits (Cambridge Bioscience, UK) according to the manufacturer's instructions. DNA was extracted from 200 mg of luminal content and 250 μ l of homogenized mucus. An initial bead-beating step was performed using a Qiagen TissueLyser at 30 Hz for 10 min. DNA was extracted from samples serially to ensure that storage time was equal for each time point. At each extraction, two controls were included: a blank extraction to control for contamination and 75 μl of Zymobiomics Standard Bacterial Community (Cambridge Bioscience, UK) to control for variations in DNA extraction efficacy. Extracted DNA was quantified using a NanoDrop 2000 spectrophotometer (NanoDrop Technologies) and a Qubit dsDNA HS fluorometric kit (Invitrogen).

### 2.4. Illumina MiSeq Sequencing

Extracted DNA was sent for paired-end sequencing of the 16S rRNA gene at the Centre for Genomic Research (University of Liverpool) using an Illumina MiSeq run. The V4 hypervariable region (515F/R806) was amplified to yield an amplicon of 254 base pairs (31). Library preparation was performed using a universal tailed tag design with subsequent amplification performed using a two step PCR with a HiFi Hot Start polymerase (Kapa) (32). The first round of PCR was performed using the primers 5'-ACACTCTTTCCCTACACGACGCTCTTCCGATCTNNNNNGTGCCAGCMGCCGCGGTAA-3' (forward) and 5'-GTGACTGGAGTTCAGACGTGTGCTCTTCCGATCTGGACTACHVGGGTWTCTAAT-3' (32). The raw Fastq files were trimmed for the presence of Illumina adapter sequences using Cutadapt version 1.2.1. The reads were further trimmed using Sickle version 1.200 with a minimum window quality score of 20. Reads shorter than 10 base pairs after trimming were removed. Raw sequence reads are available in the NCBI Sequence Repository Archive under the accession number SRP158778. A total of 2,670,876 reads were obtained from 30 experimental samples submitted for sequencing. After filtering, merging of paired reads and chimera removal, a total of 1,235,836 reads remained (46% of the original total) giving a mean of 41,194 reads per sample. The median number of reads per sample was 40,281.

### 2.5. Amplicon Sequence Variant Identification and Taxonomy Assignment

QIIME2 version 2018.4.0 was used for analysis of the Illumina data (33). Amplicon sequence variant (ASV) assignment was completed using the dada2 plugin (34) and a ASV table produced using the feature-table plugin (35). The resulting ASV table was divided into two tables: one containing all pooled samples to observe development of the ileal microbiome and one containing pooled samples from 14 d.p.h onwards to analyse differences between mucus and lumen microbiota. Taxonomy was assigned using a pre-trained NaiveBayes classifier based on the SILVA 132 database of the 515F/R806 region of the 16S rRNA gene (36) available for download at https://docs.qiime2.org/2018.11/data-resources/ using the q2-feature-classifier plugin (37).

### 2.6. Data Analysis and Statistics

Alpha and beta diversity analyses were performed at a sampling depth of 16,000 using the alignment (38), phylogeny (39), and diversity (https://github.com/qiime2/q2-diversity) plugins. While this sampling depth excluded the Hubbard mucus sample at 14 d.p.h, the increased sampling depth was considered less likely to exclude rarer taxa. Alpha diversity, a metric used to assess species richness, was measured using Faith's phylogenetic diversity (FPD) index (a measure of species richness) and a Shannon diversity (SD) index (a measure of species evenness) and compared between samples using a Kruskal Wallis test with a false discovery rate (FDR) correction. Taxa plots were produced using the q2-taxa plugin (https://github.com/qiime2/q2-taxa). Beta diversity, a metric used to compare species diversity and abundance between samples, was calculated with weighted and unweighted UniFrac metrics. The beta diversity matrix was used to draw principal coordinate analysis (PCoA) plots and an ANOSIM test was used to determine the significance of differences in beta diversity between groups.

Gneiss analysis was chosen to analyse differential abundance between groups since it overcomes challenges created by the compositional nature of microbiota data. Firstly, a dendrogram of ASVs is prepared using correlation clustering. Each node in the dendrogram is treated as a “balance” with taxa on one side of the balance termed numerators and on the other, denominators. Gneiss analysis examines the log ratio of abundances between numerator and denominator taxa at each balance. Each log ratio's final numerical value is dependent on the balance between the taxa composing the numerator and those composing the denominator of the ratio. Differences in the log ratio of a balance can be compared between sample groups to determine differences in microbiota composition. A significant difference between samples allows hypotheses to be formulated regarding changes in the absolute abundance of numerator and denominator taxa but gives no further information as to which hypothesis is correct. For example, if balance y0 is found to be significantly lower at Time A compared to Time B the following hypotheses could explain the result: (i) The numerator taxa have increased between times A and B; (ii) The denominator taxa have an decreased between times A and B; (iii) A combination of hypotheses (i) and (ii); (iv) Both numerator and denominator taxa have increased between times A and B, but numerator taxa have increased more; (v) Both numerator and denominator taxa have decreased between times A and B, but denominator taxa have decreased more. Further investigations, such as quantitative PCR, are required to discern which hypothesis is correct (40).

Gneiss analysis (40) was run using QIIME2 to identify taxa which were differentially abundant between time points and area sampled. Principal balances for use in Gneiss were obtained via Ward's hierarchical clustering using the correlation-clustering command producing a dendrogram with 200 balances (y0–y199). Isometric log ratios for each balance were calculated using the ilr-transform command. A multivariate response linear regression model of log ratios balances was constructed with area, breed and days post-hatch as covariates using the ols-regression command. Results were visualized through a regression summary and dendrogram heatmaps. Balances significantly affected by the covariates “days post hatch” and “area” were identified as those with a *p*-value < 0.05.

### 2.7. Power Calculations

Statistical power for comparisons between days post-hatch and area sampled were assessed retrospectively. ASV abundances were modeled using a Dirichlet-Multinomial model as previously described (41). Stratification by sample metadata was applied. Statistical power was calculated under this model using a Monte Carlo approach with 1,000 replications. A Wilcoxon-Mann-Whitney test was used to assess the average number of rejections of the null-hypothesis among the Monte Carlo-generated datasets to give an indication of statistical power as previously described (42). Dirichlet-Multinomial distribution modeling and power calculations were implemented using the a web interface available at https://fedematt.shinyapps.io/shinyMB.

## 3. Results

### 3.1. Statistical Power

For area sampled, in which there were 12 samples per group, a Wilcoxon-Mann-Whitney test returned a value of 0.221. A sample size of 49 samples per group would have been required to achieve a power of 0.8. For days post-hatch, in which there were 6 samples per group, in which there were 6 samples per group, a Wilcoxon-Mann-Whitney test returned a value of 0.04. A sample size of 26 samples per group would have been required to achieve a power of 0.8. These results suggest that the study was significantly underpowered increasing the probability of a Type II error. The results presented below should be interpreted in light of this finding of weak statistical power and could be regarded as more observational in nature.

### 3.2. Succession in the Ileal Microbiome

#### 3.2.1. Alpha Diversity and Beta Diversity

**Figure 1:**
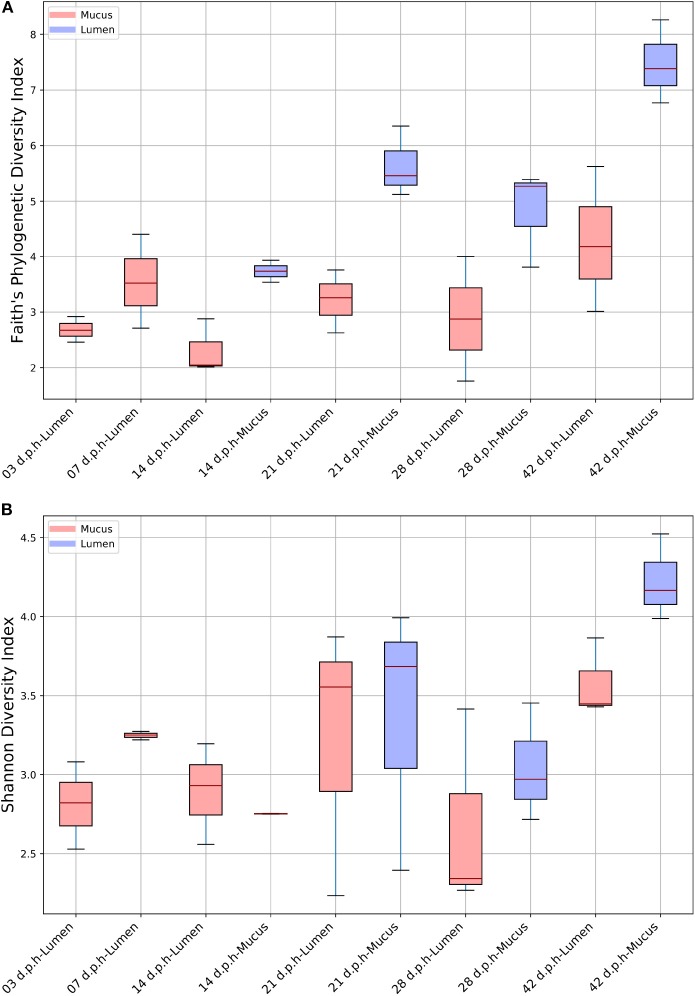
Alpha diversity in the ileum from 3 to 42 d.p.h measured by Faith's phylogenetic diversity **(A)** and Shannon diversity indices **(B)**.

Overall, age had a significant effect on alpha diversity when measured using an FPD (H = 11.4, *p* = 0.044) and SD index (H = 12.6, *p* = 0.027). Although there was an increase in alpha diversity at 42 d.p.h (Figure 1) there were no significant differences found using pairwise comparisons of alpha diversity between time points. Overall, there was no significant effect of breed on FPD (H = 2.83, *p* = 0.24) or SD index (H = 2.12, *p* = 0.35).

**Figure 2:**
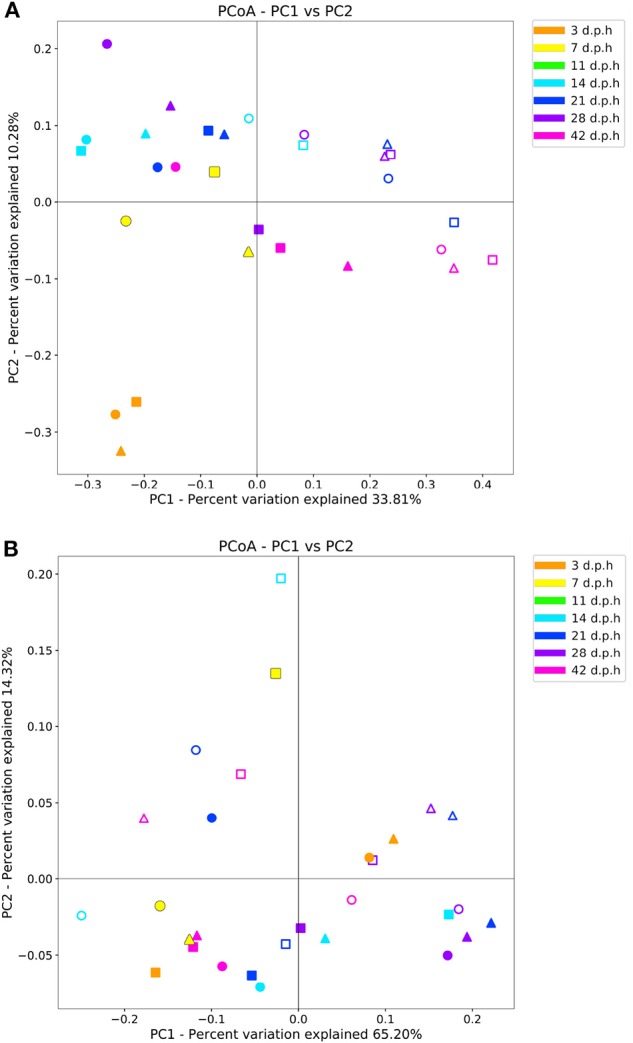
Beta diversity measured by unweighted **(A)** and weighted **(B)** UniFrac metrics in the ileum from 3 to 42 d.p.h. Samples from Hubbard (▴), Ross (■), and Cobb (•) chickens are shown with lumen (filled) and mucus (no fill) samples denoted by color fill.

Days post-hatch had a significant effect on beta diversity when measured with an unweighted and weighted UniFrac metric (*R* = 0.30, *p* = 0.002 and *R* = 0.25, *p* = 0.008, respectively). When observed using an unweighted UniFrac metric, samples from 3 d.p.h formed a distinct cluster away from other samples. From 7 d.p.h, there was no apparent pattern of clustering by time point with samples from all time points mixing together (Figure 2). When observed using a weighted UniFrac metric, there was no clustering pattern by time point. Breed had no significant effect on beta diversity when measured with an unweighted and weighted UniFrac metric (*R* = 0.02, *p* = 0.6 and *R* = 0.02, *p* = 0.6, respectively).

#### 3.2.2. Taxonomic Composition

**Figure 3:**
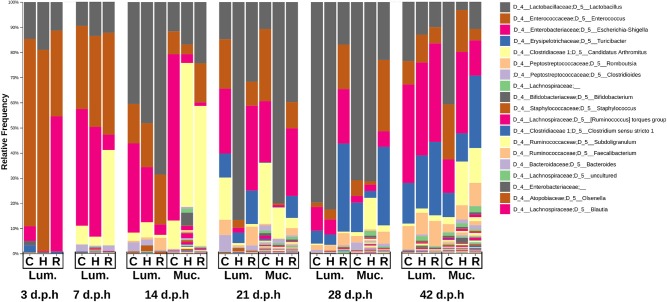
Taxa plot showing the relative abundance of bacterial genera in the ileal mucus (Muc.) and lumen (Lum.) between 3 and 42 d.p.h in Hubbard (H), Cobb (C), and Ross (R).

The composition of the microbiome at different time points was observed in a taxa plot (Figure 3). At 3 d.p.h, Enterococcaceae was the most abundant taxa in Cobb and Hubbard (74.4 and 80.1%, respectively) while Enterobacteriaceae was the most abundant in Ross (53.7%). Lactobacillaceae was present in all three breeds (Cobb = 14.7%, Hubbard = 19.0%, Ross = 11.3%). Small proportions of Bifidobacteriaceae (Cobb = 1.6%, Hubbard = 0.22%, Ross = 0.18%) and Clostridiaceae 1 (Cobb = 2.8%, Hubbard = 0.13%, Ross = 0.6%), further classified as *Clostridium sensu stricto* 1 at the genus level, were found in all three breeds.

At 7 d.p.h, the relative abundance of Lactobacillaceae (Cobb = 10.0%, Hubbard = 14.0%, Ross = 12.4%) and Enterococcaceae (Cobb = 33.1%, Hubbard = 36.1%, Ross = 40.5%) were similar between breeds. There was a higher relative abundance of Enterobacteriaceae in Cobb and Hubbard compared to Ross (Cobb = 46.4%, Hubbard = 43.7%, Ross = 6.2%) while Clostridiaceae 1, further classified as Candidatus *Arthromitus* at the genus level, was more abundant in Ross (Cobb = 7.6%, Hubbard = 3.6%, Ross = 37.9%). Peptostreptococcaceae was also present in all three breeds (Cobb = 2.7%, Hubbard = 0.57%, Ross = 2.2%).

At 14 d.p.h, Lactobacillaceae was the most abundant taxa in the lumen (Cobb = 40.5%, Hubbard = 48.2%, Ross = 68.6%). In ileal mucus, the most abundant taxa was Candidatus *Athromitus* in Ross (55.7%) and Hubbard (57.2%) with Enterobacteriaceae the most abundant in Cobb ileal mucus (66.1%). Peptostreptococcaceae (Cobb = 4.2%, Hubbard = 3.1%, Ross = 5.3%) and Staphylococcaceae (Cobb = 0.59%, Hubbard = 2.3%, Ross = 0.77%) were present in the lumen.

At 21 d.p.h, Ross and Cobb samples had a similar composition with Lactobacillaceae (Cobb = 14.8%, Ross = 31.7%), Enterococcaceae (Cobb = 19.7%, Ross = 9.5%), Enterobacteriaceae (Cobb = 25.7%, Ross = 33.7%), and Erysipelotrichaceae (Cobb = 9.5%, Ross = 13.3%) the most abundant taxa in the lumen. Lactobacillaceae was the most abundant taxa in the Hubbard lumen (86.7%) with a lower relative abundance of Enterococcaceae (3.1%), Enterobacteriaceae (1.9%) and Erysipelotrichaceae (4.2%). The composition of the mucus was similar to that of the lumen but a higher relative abundance of Candidatus *Arthromitus* was present (Cobb = 25.0%, Hubbard = 12.4%, Ross = 4.1%). Lachnospiraceae (Cobb = 5.1%, Hubbard = 2.1%, Ross = 3.0%) and Ruminococcaceae (Cobb = 1.5%, Hubbard = 1.1%, Ross = 1.8%) were present in the mucus at higher relative abundances than previously observed.

At 28 d.p.h, Lactobacillaceae was the most abundant taxa in Cobb and Hubbard lumen samples (79.7 and 82.5%, respectively) resulting in lower relative abundances of Enterococcaceae (1.7 and 3.9%, respectively), Enterobacteriaceae (9.5 and 6.1%, respectively), and Erysipelotrichaceae (5.1 and 4.1%, respectively). Ross lumen samples had a lower relative abundance of Lactobacillaceae (16.9%) and a higher relative abundance of Enterococcaceae (17.7%), Enterobacteriaceae (21.7%), and Erysipelotrichaceae (35.0%). Peptostreptococcaceae was present in all lumen samples (Cobb = 3.1%, Hubbard = 2.1%, Ross = 5.3%). The composition of mucus samples was similar to that of lumen samples.

At 42 d.p.h, there was a similar taxonomic composition between breeds with Lactobacillaceae (Cobb = 23.5%, Hubbard = 13.1%, Ross = 10.0%), Enterococcaceae (Cobb = 9.4%, Hubbard = 11.1%, Ross = 6.5%), Enterobacteriaceae (Cobb = 39.1%, Hubbard = 37.5%, Ross = 39.2%), Erysipelotrichaceae (Cobb = 16.1%, Hubbard = 21.1%, Ross = 29.2%), and Peptostreptococcaceae (Cobb = 9.4%, Hubbard = 9.2%, Ross = 10.2%) forming the major taxa in the ileal lumen. The composition of the mucus microbiome was similar to that of the lumen but higher relative abundances of Candidatus *Arthromitus* (Cobb = 3.1%, Hubbard = 17.4%, Ross = 13.9%), Lachnospiraceae (Cobb = 3.7%, Hubbard = 3.7%, Ross = 6.6%), and Ruminococcaceae (Cobb = 2.2%, Hubbard = 3.7%, Ross = 6.6%) were observed.

#### 3.2.3. Differentially Abundant ASVs

Gneiss analysis revealed differential ASV abundance from 3 to 42 d.p.h in the ileum. The ASV table was filtered to exclude ASVs with a frequency of less than 29 reducing the number of ASVs in the analysis from 391 to 201. The overall linear regression model fit was R2 = 0.59 with covariates “07,” “14,” “21,” “28,” and “42 d.p.h” accounting for 6.3, 10.7, 10.8, 12.9, and 16.1% of variance, respectively. Log ratio balances y0, y1, y2, y6, y10, y11, and y18 were significant predictors for one or more time points.

**Figure 4:**
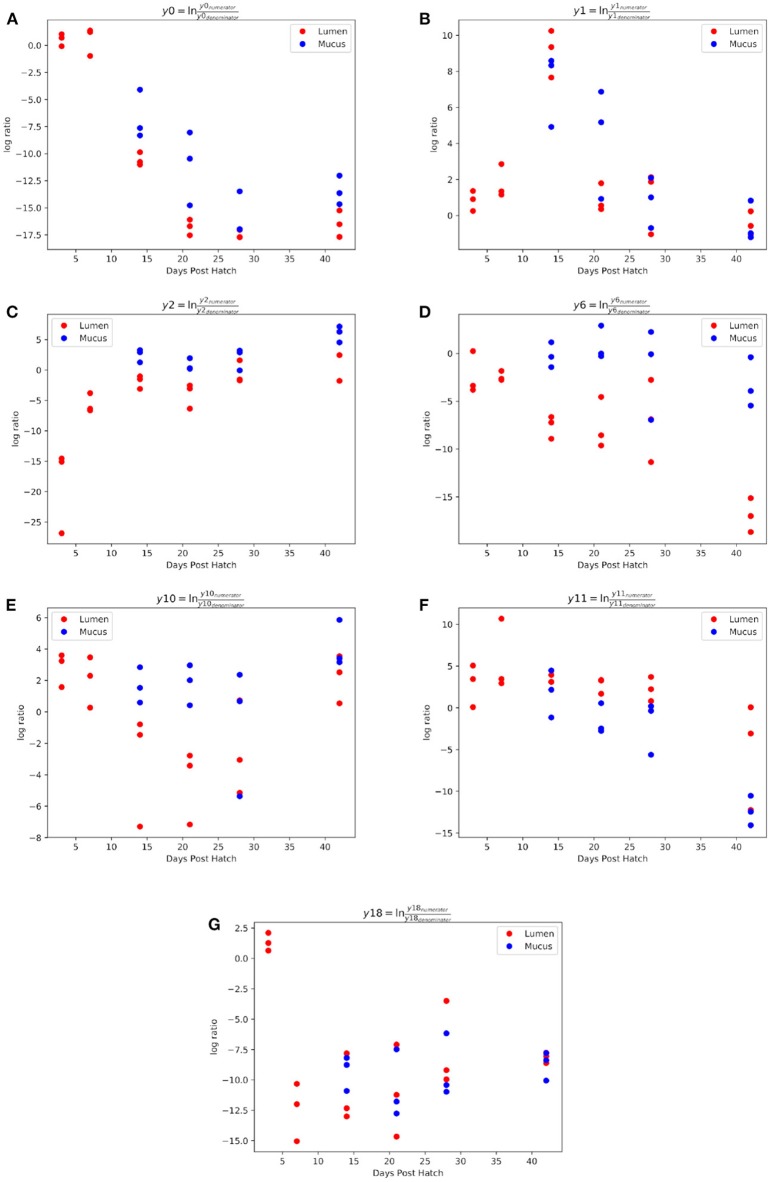
Log ratios of balances y0 **(A)**, y1 **(B)**, y2 **(C)**, y6 **(D)**, y10 **(E)**, y11 **(F)**, and y18 **(G)** which were identified as significantly different between time points during ileal microbiome development.

The log ratio of balance y0 was significantly lower at 14 (β = –10.9, *p* < 0.001), 21 (β = –16.3, *p* < 0.001), 28 (β = –19.0, *p* < 0.001), and 42 (β = –17.3, *p* < 0.001) d.p.h showing that y0_denominator_ ASVs were associated with a later microbiome (Figure 4A). Balance y1, a subdivision of balance y0_denominator_ ASVs, was significantly higher at 14 d.p.h (β = 7.2, *p* < 0.001) (Figure 4B), allowing y0_denominator_ ASVs to be separated into those that were present at 14 d.p.h (y1_numerator_) and those present from 21 d.p.h (y1_denominator_).

**Figure 5:**
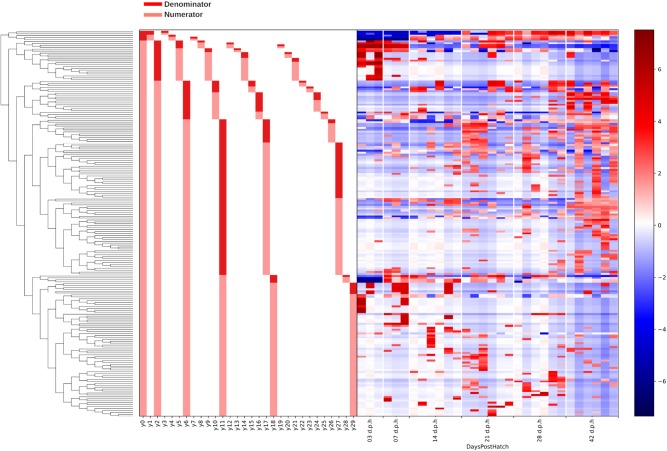
A dendrogram heatmap showing the log abundance of ASVs in ileal lumen and mucus samples grouped by age. Differences in relative abundance between the time points are visible in balances y0, y1, y2, y6, y10, y11, and y18 identified by Gneiss analysis as containing differentially abundant ASVs.

Balance y2 is a subdivision of y0_numerator_ ASVs. The log ratio of balance y2 was significantly higher at 7 (β = 13.2, *p* < 0.001), 14 (β = 17.2, *p* < 0.001), 21 (β = 15.3, *p* < 0.001), 28 (β = 17.6, *p* < 0.001), and 42 (β = 21.1, *p* < 0.001) d.p.h (Figure 4C). The dendrogram heatmap (Figure 5) shows that this is due to an increased log abundance of y2_denominator_ ASVs at 3 d.p.h and increasing log abundance of y2_numerator_ ASVs at later time points.

Balance y6 is a subdivision of y2_numerator_ ASVs. The log ratio of balance y6 was significantly lower at 42 d.p.h (β = –12.2, *p* = 0.002) with the dendrogram heatmap showing this is due to an increased log abundance of y6_denominator_ ASVs at this time point (Figure 4D). Balance y10 is a subdivision of y6_denominator_ ASVs and had a significantly lower log ratio at 14 (β = –5.4, *p* = 0.02), 21 (β = –6.0, *p* = 0.01), and 28 (β = –6.3, *p* = 0.008) d.p.h (Figure 4E). The dendrogram heatmap shows the lower log ratio was produced by an increased log abundance of y10_denominator_ ASVs at those time points.

Balance y11 is a subdivision of y6_numerator_ ASVs. The log ratio of balance y11 was significantly lower at 42 d.p.h (β = –9.4, *p* = 0.002) (Figure 4F). The dendrogram heatmap shows that y11_denominator_ ASVs were more abundant at 42 d.p.h, although some were also present at earlier time points. Balance y18 is a subdivision of y11_numerator_ ASVs. The log ratio of balance y18 was significantly lower at 7 (β = –13.8, *p* < 0.001), 14 (β = –11.5, *p* < 0.001), 21 (β = –12.2, *p* < 0.001), 28 (β = –9.7, *p* < 0.001), and 42 (β = –9.8, *p* < 0.001) d.p.h (Figure 4G). The dendrogram heatmap shows that this lower value was caused by a higher log abundance of y18_denominator_ ASVs. The log abundance of y18_numerator_ ASVs was sporadic and not related to any time point in particular.

Taxonomic classification of ASVs in balances is presented in Table 2. The results of Gneiss analysis mirror what was described by the taxa plots, however, some more details can be discerned. ASVs assigned to Lactobacillaceae were identified as differentially abundant at 3, 14, and 42 d.p.h. At the species level, Lactobacillaceae ASVs present at 3 d.p.h were classified as *Lactobacillus mucosae* whilst those from later time points were assigned to *Lactobacillus vaginalis* or were not assigned further than the genus *Lactobacillus*. ASVs assigned to Erysipelotrichaceae were identified as differentially abundant at 0 d.p.h and 28 and 42 d.p.h. At lower taxonomic levels, those differentially abundant at 0 d.p.h were assigned to *Erysipelatoclostridium* while those at 28 and 42 d.p.h were assigned to *Turicibacter*. Another family which was identified as differentially abundant at multiple time points was Peptostreptococcaceae, with two ASVs abundant at 7 d.p.h, four at 14 d.p.h and two at 42 d.p.h. As with Lactobacillaceae, greater taxonomic resolution revealed that Peptostreptococcaceae ASVs colonizing at 7 d.p.h were assigned to the genus *Clostridioides* while later colonizers were assigned to *Romboutsia*. ASVs assigned to Clostridiaceae 1 at 7 d.p.h were further classified as Candidatus *Arthromitus* but those identified as colonizing at 3 and 42 d.p.h were assigned to *Clostridium sensu stricto* 1 at the genus level.

**Table 2:** Taxonomy of ASVs colonizing the ileum at different time points.

		**Number of ASVs**					
**Taxonomy**	**Total count**	**3 d.p.h**	**7 d.p.h**	**14 d.p.h**	**21 d.p.h**	**28 & 42 d.p.h**	**NDA^a^**
D_4__Lachnospiraceae	54	1	0	0	0	39	14
D_4__Ruminococcaceae	40	0	0	0	0	28	12
D_4__Enterococcaceae	23	13	0	0	0	1	9
D_4__Enterobacteriaceae	16	2	0	0	0	4	10
D_4__Peptostreptococcaceae	12	0	2	4	0	2	4
D_4__Lactobacillaceae	10	2	0	2	0	4	2
D_4__Clostridiaceae 1	9	1	2	0	0	2	4
D_4__Corynebacteriaceae	4	0	0	1	0	0	3
D_4__Staphylococcaceae	4	0	0	2	0	0	2
D_4__Erysipelotrichaceae	3	0	0	0	2	1	0
D_4__Peptococcaceae	3	0	0	0	0	3	0
D_4__Christensenellaceae	2	0	0	0	0	0	2
D_4__Aerococcaceae	2	0	0	0	0	1	1
D_4__Burkholderiaceae	2	0	0	0	0	1	1
D_4__Bacteroidaceae	2	0	0	0	0	2	0
D_4__Streptococcaceae	2	1	0	0	0	1	0
D_4__Bacillaceae	2	1	0	0	0	1	0
D_4__Campylobacteraceae	2	0	0	0	0	1	1
D_4__Dermabacteraceae	2	0	0	0	0	0	2
D_4__Planococcaceae	1	0	0	0	0	1	0
D_4__Pseudomonadaceae	1	0	0	0	0	0	1
D_4__Atopobiaceae	1	0	0	0	0	1	0
D_4__Bifidobacteriaceae	1	0	0	0	0	0	1
D_4__Rhizobiaceae	1	0	0	0	0	0	1
D_4__Coriobacteriaceae	1	0	0	0	0	1	0
D_4__Paenibacillaceae	1	0	0	0	0	1	0

a *ASVs defined as NDA were not assigned a time point at which colonization was significant. Individual taxonomies of significant Gneiss balances are provided in Supplementary Figure 1*.

### 3.3. Differences Between the Lumen and Mucus Microbiomes

Samples taken between 14 and 42 d.p.h were used to analyse differences between mucus and lumen microbiomes. As before, sequencing depth was set at 16,000 for diversity analyses excluding the Hubbard mucus sample at 14 d.p.h.

#### 3.3.1. Alpha Diversity and Beta Diversity

For alpha diversity, samples were grouped by area sampled and days post-hatch. When measured using a FPD index, there was a significant of area sampled and days post-hatch on alpha diversity (H = 17.8, *p* = 0.01). Although there were no significant differences in alpha diversity between lumen and mucus samples at the sample time point, a plot of FPD alpha diversity shows that mucus samples tended to have a higher alpha diversity than lumen samples (Figure 1). However, there was no overall effect of area sampled and days post-hatch on alpha diversity when measured using a SD index (H = 11.1, *p* = 0.13). In this case, alpha diversity plots showed no differences between mucus and lumen samples at 14 and 21 d.p.h, although mucus samples had a higher average alpha diversity at 28 and 42 d.p.h (Figure 1). This indicates that the differences in FPD alpha diversity were largely driven by low abundance ASVs.

When measured using an unweighted UniFrac metric, there was a significant impact of area sampled on beta diversity (*R* = 0.63, *p* = 0.001). However, a significant effect of area sampled was not observed when a weighted UniFrac metric was used (*R* = 0.05, *p* = 0.16). A PCoA plot of unweighted UniFrac beta diversity show mucus samples cluster apart from lumen samples (Figure 2) with no clustering pattern visible in the PCoA plot of weighted UniFrac beta diversity (Figure 2). These results suggest that while the composition of the mucus microbiome is different, the relative abundance of the most common ASVs is similar between the mucus and lumen.

#### 3.3.2. Differentially Abundant ASVs

Gneiss analysis revealed differential ASV abundance from 3 to 42 d.p.h in the ileum. The ASV table was filtered to exclude ASVs with a frequency of less than 29 reducing the number of ASVs in the analysis from 343 to 178. The overall linear regression model fit was R2 = 0.37 with covariate “Area” accounting for 14.7% of variance. Log ratio balances y2 (β = –9.6, *p* < 0.001), y7 (β = 5.2, *p* = 0.004), y8 (β = 4.9, *p* = 0.03), y26 (β = –3.9, *p* = 0.04), and y30 (β = 3.4, *p* = 0.04) were significant predictors for “Area.”

**Figure 6:**
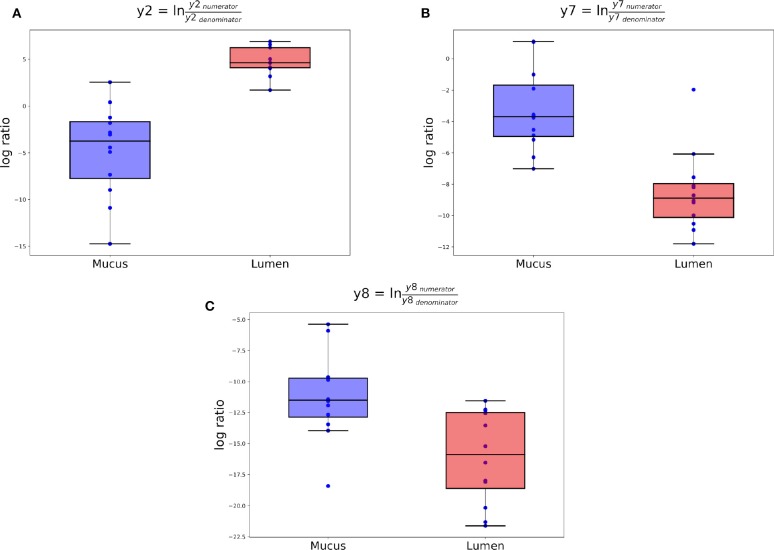
Log ratios of balances y2 **(A)**, y7 **(B)**, and y8 **(C)** that were identified as significantly different between ileal lumen and mucus samples.

**Figure 7:**
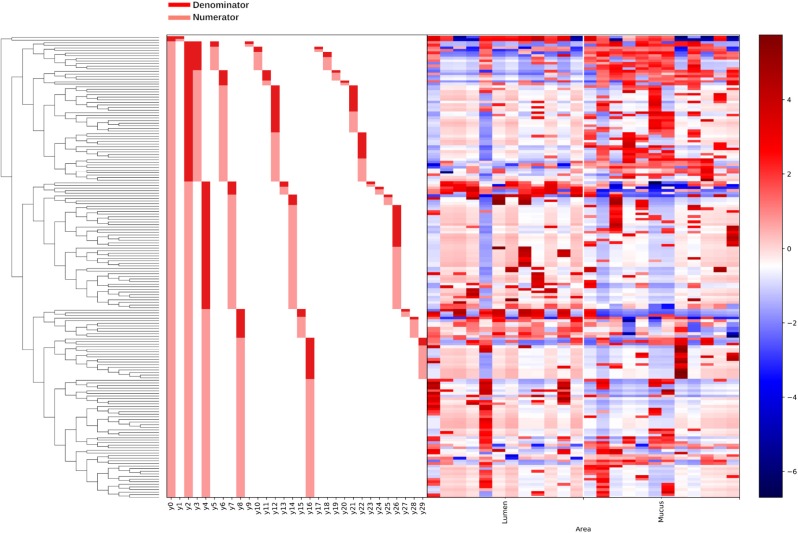
A dendrogram heatmap showing the log abundance of ASVs in ileal lumen and mucus samples grouped by age. Differences in relative abundance are visible between the time points in balances y2, y7, y8 identified by Gneiss analysis as containing differentially abundant ASVs.

The log ratio of balance y0 was not significantly affected by “Area” allowing the conclusion that y0_denominator_ ASVs were not differentially abundant between mucus and lumen samples. Balance y2 is a subdivision of y0_numerator_ ASVs and was significantly lower in mucus samples (Figure 6A). The dendrogram heatmap (Figure 7) shows that this was due to an increased relative abundance of y2_denominator_ ASVs in mucus samples.

Balance y4 is a subdivision of y2_numerator_ ASVs but the log ratio of this balance was not significantly different between mucus and lumen samples. Balance y7 is a subdivision of y4_denominator_ ASVs and was significantly lower in lumen samples (Figure 6B). The dendrogram heatmap shows that this was due to an increased relative abundance of y7_denominator_ ASVs in lumen samples while y7_numerator_ ASVs were similarly abundant in mucus and lumen samples. Balance y8 is a subdivision of y4_numerator_ ASVs and was significantly lower in lumen samples (Figure 6C). The dendrogram heatmap shows that this was due to an increased relative abundance of y8_denominator_ ASVs in lumen samples while y8_numerator_ ASVs were similarly abundant in mucus and lumen samples.

Taxonomic classification of ASVs in balances is presented in Table 3. Most ASVs showed no preference for colonizing either the mucus or the lumen. Most ASVs identified as more abundant in the mucus were classified as Lachnospiraceae and Ruminococcaceae. Three ASVs assigned to Clostridiaceae 1 were more abundant in mucus. All three of these ASVs were assigned to Candidatus *Arthromitus* at the genus level. The remaining four Clostridiaceae 1 ASVs included in the analysis were classified as not differentially abundant between mucus and lumen samples and were all assigned to *Clostridium sensu stricto* 1 at the genus level. Some taxa which were previously identified as more abundant in the caecum had a higher relative abundance in the mucus including Peptococcaceae, Bacteroidaceae, Burkholderiaceae, Christensenellaceae, and Bacillaceae. Nine ASVs assigned to Lactobacillaceae were included in the analysis. Of these, one was assigned to *Lactococcus*, two to *Lactobacillus mucosae*, three to *Lactobacillus vaginalis*, and four to *Lactobacillus*. Both ASVs assigned to *Lactobacillus mucosae* were classified as more abundant in the lumen. 12 ASVs assigned to Peptostreptococcaceae were included in the analysis. Of these, six were assigned to *Romboutsia* and six to *Clostridioides* at the genus level. A total of five ASVs assigned to Peptostreptococcaceae were classified as more abundant in the lumen. At the genus level, four of these ASVs were assigned to *Romboutsia* with the remaining ASV assigned to *Clostridioides*.

**Table 3:** Taxonomy of ASVs identified as differentially abundant between mucus and lumen samples in the ileum.

		**Number of ASVs**		
**Taxonomy**	**Total**	**Lumen**	**Mucus**	**NDA^a^**
D_4__Lachnospiraceae	53	0	18	35
D_4__Ruminococcaceae	40	0	23	17
D_4__Enterobacteriaceae	14	2	0	12
D_4__Peptostreptococcaceae	12	5	0	7
D_4__Lactobacillaceae	9	2	1	6
D_4__Enterococcaceae	9	4	0	5
D_4__Clostridiaceae 1	7	0	3	4
D_4__Corynebacteriaceae	4	1	0	3
D_4__Staphylococcaceae	4	2	0	2
D_4__Erysipelotrichaceae	3	0	0	3
D_4__Peptococcaceae	3	0	2	1
D_4__Bacteroidaceae	2	0	2	0
D_4__Burkholderiaceae	2	0	1	1
D_4__Christensenellaceae	2	0	2	0
D_4__Campylobacteraceae	2	0	1	1
D_4__Streptococcaceae	2	0	0	2
D_4__Dermabacteraceae	2	0	0	2
D_4__Aerococcaceae	2	0	0	2
D_4__Paenibacillaceae	1	0	0	1
D_4__Planococcaceae	1	0	0	1
D_4__Atopobiaceae	1	0	0	1
D_4__Bifidobacteriaceae	1	0	0	1
D_4__Coriobacteriaceae	1	0	0	1
D_4__Bacillaceae	1	0	1	0

a *ASVs defined as NDA were not differentially abundant between ileal mucus and lumen samples. Individual taxonomies of significant Gneiss balances are provided in Supplementary Figure 2*.

## 4. Discussion

Power calculations conducted retrospectively indicate that this study was significantly underpowered and the results presented above must be interpreted in light of this finding. As a result, findings presented should be interpreted as observational with poor statistical support. Despite this caveat of small sample size reducing statistical power, some patterns within the data merit discussion and may provide the basis for further research with adequate sample sizes.

### 4.1. General Pattern of Succession

The results presented above describe a pattern of succession in the ileal microbiome undergoing several shifts in taxonomic composition before a mature community is present. Most studies observing bacterial succession in the ileum produce differing results. Early microbiomes are the most variable between studies. This is likely due to different bacterial exposure between hatcheries (23). Most agree that this initial community is replaced by a rise in the abundance of Lactobacillaceae (28, 43, 44) however, the timing of this rise often differs between studies. Many factors are likely to contribute to these discrepancies such as environmental exposure, diet, and differences in methodology. Stanley et al. (45) showed significant differences in intestinal microbiota between three trials even though the chickens were kept under the same conditions and fed the same diet. There is also no agreement as to whether Lactobacillaceae remains the dominant taxa within the microbiome. Some have found no difference in abundance at time points from 8 to 36 d.p.h while others have observed a decrease in Lactobacillaceae between 4 and 16 d.p.h (28, 46).

A similar pattern of succession to that described in this study has been proposed by Jurburg et al. (25) after examining the fecal microbiome of chickens between 1 and 35 d.p.h although many taxa detected are associated with the caecal microbiome rather than the ileal microbiome. The first stage was dominated by rapidly-colonizing taxa such as *Streptococcus* and *Escherichia/Shigella*. *Lactobacillus* became more prominent in the fecal microbiome from 3 d.p.h with an increase in relative abundance at 14 d.p.h. The peak of Candidatus *Arthromitus* abundance was noted at 14 and 21 d.p.h. Slower growing taxa such as *Romboutsia* and other Peptostreptococcaceae colonized from 21 d.p.h. The similarities between these results and those described above support the conclusion that the ileal microbiome undergoes several shifts in composition from hatch to 35 and 42 d.p.h. Further evidence that the ileal microbiome isn't stable until later time points can be found in the results of those using techniques other than next generation sequencing. Lu et al. (16), found that clone libraries at 3, 7, and 49 d.p.h had a high dissimilarity to those from 14, 21, and 28 d.p.h with *Lactobacillus* decreasing while *Clostridia* increased over time. Similarly, den Hartog et al. (47) used T-RFLP reads to demonstrate a temporary disturbance in ileal microbiome composition between 14 and 42 d.p.h.

I am inclined to conclude that high Lactobacillaceae abundance is not the hallmark of a mature ileal microbiome but rather a stage of maturation. Much work has been done to characterize the bacterial composition of the ileal microbiome in chickens at fixed time points. Many of these focus on the microbiome between 21 and 35 d.p.h, presumably under the impression that the ileal microbiome has matured by this point. Indeed, Amit-Romach stated in 2004 that “A typical microflora of adult birds in the small intestine is established within 2 weeks” (48). This has perhaps led to the assumption that a mature ileal microbiome is dominated by Lactobacillaceae.

Although the general pattern of succession followed that described by previous studies, examination of Lactobacillaceae and Peptostreptococcaceae ASVs at the genus and species level revealed a more detailed pattern of succession. Most importantly, ASVs assigned to *Lactobacillus mucosae* colonized the ileum earlier than those assigned to other species of Lactobacillaceae. Although the order of succession in the ileal microbiome may simply be a matter of presence or absence of taxa it is possible that some strains of bacteria are better adapted to colonize the early gut whilst others require alterations to the gut environment produced by early inhabitants before they are able to colonize. While more detailed studies considering bacterial succession using identification at higher taxonomic levels would be required to confirm this hypothesis, it is worth considering in terms of selection of potential probiotic species. Currently, probiotics may be selected due to *in vitro* activity against pathogens, production of certain metabolites or suitability to industrial production and administration. However, the ability of strains to colonize the gut immediately post-hatch would also influence the functional success of a probiotic product.

This study should also highlight two groups of ileal bacteria that have previously been neglected but which may be of interest. Peptostreptococcaceae is often reported in the ileum but eclipsed by Lactobacillaceae. Firstly, the ASVs assigned to Peptostreptococcaceae in this study were further identified as *Clostridioides* and *Romboutsia* with *Clostridioides* colonizing the ileum from 7 d.p.h and *Romboutsia* present from 14 d.p.h. The human pathogen *Clostridium difficile* was reclassified in 2016 to the genus *Clostridioides* (49). The two ASVs assigned to *Clostridioides* in this study were not classified to the species level by comparison against the SILVA database. An search of the NCBI bacterial 16S rRNA gene database using BLAST revealed 98.13 and 98.51% similarity to sequences from *Clostridioides difficile*. The presence of *Clostridioides difficile* in the chicken ileum has been reported and discussed with respect to public health (50) but no studies mention the impact of this species on poultry health. Genomic and functional analysis of *Romboutsia* found in the human gastrointestinal tract reveal that these Clostridia are highly adapted to life in the small intestine with the ability to ferment glucose and other simple carbohydrates (51). Secondly, Erysipelotrichaceae was found in the ileum from 21 d.p.h in this study. These ASVs were classified as *Turicibacter* at the genus level. Studies of *Turicibacter* metabolism and functional genomics are not yet available, however, the abundance of *Turicibacter* in this study should highlight it as another neglected but important member of the mature ileal microbiome.

### 4.2. Differences in Succession Between Breeds

When describing differences in succession between breeds using the results described above, the limitations of sample size and pooling should be considered. Small sample size reduces the ability to support observations with statistical tests. As such, the description of differences in succession between breeds is purely observational and without statistical support. Equally, pooling samples masks individual variation between chickens. It is possible that pooled samples were more representative of certain constituent samples than others.

The general pattern of succession described above was followed by all breeds with the exception that no sudden rise in Candidatus *Arthromitus* abundance was observed in Cobbs. However, the rate of succession differed between breeds. Ross were the first to demonstrate sequential rises in Enterobacteriaceae (3 d.p.h), Candidatus *Arthromitus* (7 d.p.h), Lactobacillaceae (14 d.p.h), and Erysipelotrichaceae (28 d.p.h). This pattern was followed, first by Hubbard and then by Cobb. The peak in Candidatus *Arthromitus* abundance was visible in Hubbards at 14 d.p.h and may have occurred in Cobb between 14 and 21 d.p.h. Lactobacillaceae abundance rose in Hubbard at 21 d.p.h and Cobb at 28 d.p.h. The dominance of Lactobacillaceae was more sustained in Hubbard as it was still observable 7 days later at 28 d.p.h. An increase in Enterobacteriaceae and Erysipelotrichaceae with a concurrent decrease in Lactobacillaceae was apparent in both Cobb and Hubbard by 42 d.p.h.

Three previous studies provide direct comparisons between Ross, Cobb, and Hubbard. They focus on the responses of the three breeds to necrotic enteritis. In general, they have found that Cobb are more susceptible to necrotic enteritis than Ross and Hubbard, exhibiting increased body weight loss and more severe intestinal lesions (52). Hubbard appear to have an intermediary susceptibility to necrotic enteritis with Ross emerging as the most resistant. In one study, Hubbard chicks lost more body weight than Ross chicks but did not have more severe intestinal lesions (53). In another, Hubbard chicks showed no significant loss of body weight compared to Ross chicks and showed an intermediate severity of intestinal lesions between Ross and Cobb chicks (52). Ross have been shown to have differential expression of β-defensin genes during necrotic enteritis infection when compared to Cobb (54). These results are interesting in light of the observation above that Ross had an accelerated development of the ileal microbiome when compared to Cobb, with Hubbard as an intermediary.

The protocol for inducing necrotic enteritis in all three experiments was the same. *Eimeria maxima* oocyts were administered at 14 d.p.h followed by *Clostridium perfringens* at 18 d.p.h (52–54). It follows that any difference in response to necrotic enteritis should have its roots during the first 2 weeks of life. The most evident difference between the three breeds during this period was the appearance of Candidatus *Arthromitus*. This genus formed a great part of the microbiome in Ross chicks from 7 to 14 d.p.h and Hubbard at 14 d.p.h, but was less abundant in Cobb. It is possible that this lack of early colonization by Candidatus *Arthromitus* could leave Cobb chickens more susceptible to infectious disease as the interaction between Candidatus *Arthromitus* and host tissue plays a role in immune maturation.

Stanley et al. (55) used Cobb chickens to investigate changes in caecal microbiota to dietary fishmeal, *Eimeria* and *C. perfringens* in a model of necrotic enteritis. The abundance of Candidatus *Arthromitus* was observed to increase in response to infection with *C. perfringens* but was not present in any groups treated with *Eimeria*. The authors suggest that *Eimeria* could remove Candidatus *Arthromitus* as a mechanism for modulating host immunity and thereby increase its own infectivity. However, the removal of Candidatus *Arthromitus* leads to an imbalance in mucosal immunity allowing *C. perfringens* to grow unchecked (55). Further weight is added to this argument by the observation that fumonisin mycotoxins in feed also lower the abundance of Candidatus *Arthromitus* and increase the susceptibility to *C. perfringens* infection and development of necrotic enteritis (56).

### 4.3. Differences Between Mucus and Lumen Microbiomes

Significant differences between the composition of the mucus and lumen microbiomes were discovered. Candidatus *Arthromitus* was identified as significantly more abundant in the mucus. This finding is likely due to Candidatus *Arthromitus*' close association with the ileal epithelium during its life cycle (57). Other taxa identified as differentially abundant in the mucus were those previously classified as associated with the caecal microbiome such as Lachnospiraceae, Ruminococcaceae, Peptococcaceae, and Bacteroidaceae. A similar result was obtained by Borda-Molina et al. (21) who found Lachnospiraceae, Ruminococcaceae, and Burkholderiaceae to be more prevalent in the crop and ileal mucosa compared to digesta. Their presence in the ileum may be due to ingestion of these bacteria from the feces of other birds or retroperistalsis from the caecum.

Some taxa were identified as differentially abundant in the ileal lumen including *Romboutsia*, two Lactobacillaceae, four Enterococcaceae and two Staphylococcaceae. In the absence of quantitative data it is not possible to discern whether the increase in relative abundance of these ASVs in the lumen was due to an absolute increase in their abundance or a decrease in the abundance of Candidatus *Arthromitus*. If a true biological preference for the lumen were present it would be expected that a majority, if not all, ASVs assigned to a taxa would be differentially abundant in that niche as in the case of Candidatus *Arthromitus*. This was not observed for Lactobacillaceae, Enterococcaceae, or Staphylococcaceae. Four of six ASVs assigned to *Romboutsia* were differentially abundant in the lumen, presenting a stronger case for a true biological preference for lumen colonization. Under laboratory conditions, *Romboutsia* is unable to grow on mucin which is likely to due its inability to degrade mucus derived carbohydrates such as L-fucose and sialic acid (51). This experimental data supports the finding that *Romboutsia* was differentially abundant in the lumen and may have a higher absolute abundance in the lumen.

### 4.4. Candidatus *Arthromitus*: Colonization and Immune Development

Candidatus *Arthromitus* is a segmented filamentous bacteria (SFB), a group of host specific, non-pathogenic bacteria which are often found associated with the terminal ileum of animals (58). In recent years the appropriate taxonomy has been debated. *Candidatus* Arthromitus was originally proposed due to the morphological similarity with bacteria associated with the arthropod intestinal tract discovered by Leidy (59) and Snel et al. (58). However, 16S rRNA gene sequencing has revealed that, despite their morphological similarity, Leidy's arthropod SFB belong to Lachnospiraceae whilst those described in vertebrates belong to Clostridiaceae (60). In light of this the name Candidatus *Savagella* has been proposed although both names are still used in the literature (60).

The life cycle of SFB is complex and involves extensive contact with the ileal epithelium but since SFB are unculturable details must be discerned from microscopy (57). SFB exist in two morphological forms, a dormant spore and an active holdfast. Some studies suggest that the holdfasts begin to grow in the lumen before developing specialized ends which invade the epithelium (61). Others hypothesize that holdfasts are initially motile and attach to the epithelium before more segments begin to grow (62). Each segment develops two intrasegmental bodies which differentiate into either spores or holdfasts which are released into the lumen (62).

Previous studies observing the colonization and distribution of SFB in the chicken gut have produced similar results to those presented above. SFB are found principally in the ileal mucus with some presence in ileal content (63) although there is one report of SFB in the caecal tonsils (64). SFB appear during the first week of life, reaching peak abundance between 9 and 14 d.p.h before declining (63, 64). The pattern of colonization varies greatly among individual birds, especially at an early age. One possible explanation is the influence of other members of the microbiome. Earlier SFB colonization was observed in chickens fed a *Lactobacillus delbrueckii* probiotic (63). Equally this variation could be explained by differing levels of maternal IgA transfer to chicks. The decline of SFB in chicks is linked to increasing concentrations of intestinal IgA (63, 65). This is similar to observations in mice in which SFB colonization doesn't occur until weaning when maternal IgA is no longer supplied to the gut and declines once endogenous IgA production reaches sufficient level (66).

All studies investigating the effect of SFB on host immune development have used comparisons between gnotobiotic and germfree mice. Any comparison between mammalian and avian immune development is likely to produce false assumptions not only because SFB appear to interact with different epithelial cells between species (57) but also because the function of some immune cell subsets in avian immunology is still unknown. However, it is worth noting that SFB have been shown to stimulate various parts of the mammalian immune system and it should be worth pursuing similar experiments in chickens to discern the role that SFB play in the development of the avian immune system. SFB have been linked to such diverse roles as the stimulation of IgA production (67–69), increasing numbers of αβ— and αα—intraepithelial T-cells (70, 71), inducing expression of fucosyl sialo GM1 glycolipids which may inhibit attachment of other bacteria (71), induction of Th17 cells and decreased regulatory T cells (57, 70).

## 5. Conclusion

The early ileal microbiome had a low diversity with Enterobacteriaceae and Enterococcaceae found to be the most abundant taxa. *Lactobacillus mucosae* was present at 3 d.p.h but other species of *Lactobacillus* such as *Lactobacillus vaginalis* did not colonize the ileum until later time points. A pattern of succession followed with Candidatus *Arthromitus* and *Clostridioides* appearing in the ileal microbiome at 7 d.p.h. Candidatus *Arthromitus* became the most abundant taxa in the mucus while Lactobacillaceae was the most abundant in the lumen. The high abundance of Candidatus *Arthromitus* was short lived as Lactobacillaceae became the most abundant genus in both the mucus and lumen. High abundance of Lactobacillaceae was a transient feature of the ileal microbiome with Peptostreptococcaceae, Enterobacteriaceae, Enterococcaceae, and Erysipelotrichaceae increasing in abundance at later time points. This general pattern of succession was followed by all breeds, however, the rate at which succession occurred was different with the intestinal microbiome of Ross birds advancing through the described pattern of succession quicker than Hubbard and Cobb. These differences in succession, especially a disparity in Candidatus *Arthromitus* abundance, could explain differences in the susceptibility to infectious enteric disease previously observed between these three breeds.

Significant differences between the lumen and mucus microbiomes were observed with Candidatus *Arthromitus* and caecal bacteria such as Lachnospiraceae, Ruminococacaeae, and Burkholderiaceae showing increased abundance in the mucus and *Romboutsia* showing increased abundance in the lumen.

## Data Availability Statement

The datasets generated for this study can be found in the NCBI Sequence Repository Archive under accession number SRP158778.

## Ethics Statement

The animal study was reviewed and approved by University of Liverpool Animal Welfare and Ethical Review Body.

## Author Contributions

PR-R conducted the sampling, sample processing, 16S rRNA gene analysis, interpretation of the results, and wrote the manuscript. MB advised on the design of the experiment and edited the manuscript. JF advised on experimental design and data analysis relating to 16S rRNA gene sequencing. PW advised on experimental design and helped conduct the experiment. All authors read and approved the final manuscript.

### Conflict of Interest

The authors declare that this study received funding from DuPont Industrial Biosciences. The funder advised on the study design and approved the final manuscript for publication but did not have a role in the data collection and analysis or decision to publish. MB was employed by DuPont Industrial Biosciences. The remaining authors declare that the research was conducted in the absence of any commercial or financial relationships that could be construed as a potential conflict of interest.
