# Supplementary material for: Development of the Ileal Microbiota in Three Broiler Breeds
Source: Front Vet Sci. 2020 Jan 30;7:17. doi: 10.3389/fvets.2020.00017 (PMC7002466; doi:10.3389/fvets.2020.00017)
Supplement: Supplementary file 1 [file Data_Sheet_1.docx]

**Supplementary Figure 1.** Barplots showing the taxonomic composition of balances which reveal significant differences between time points in the ileum.


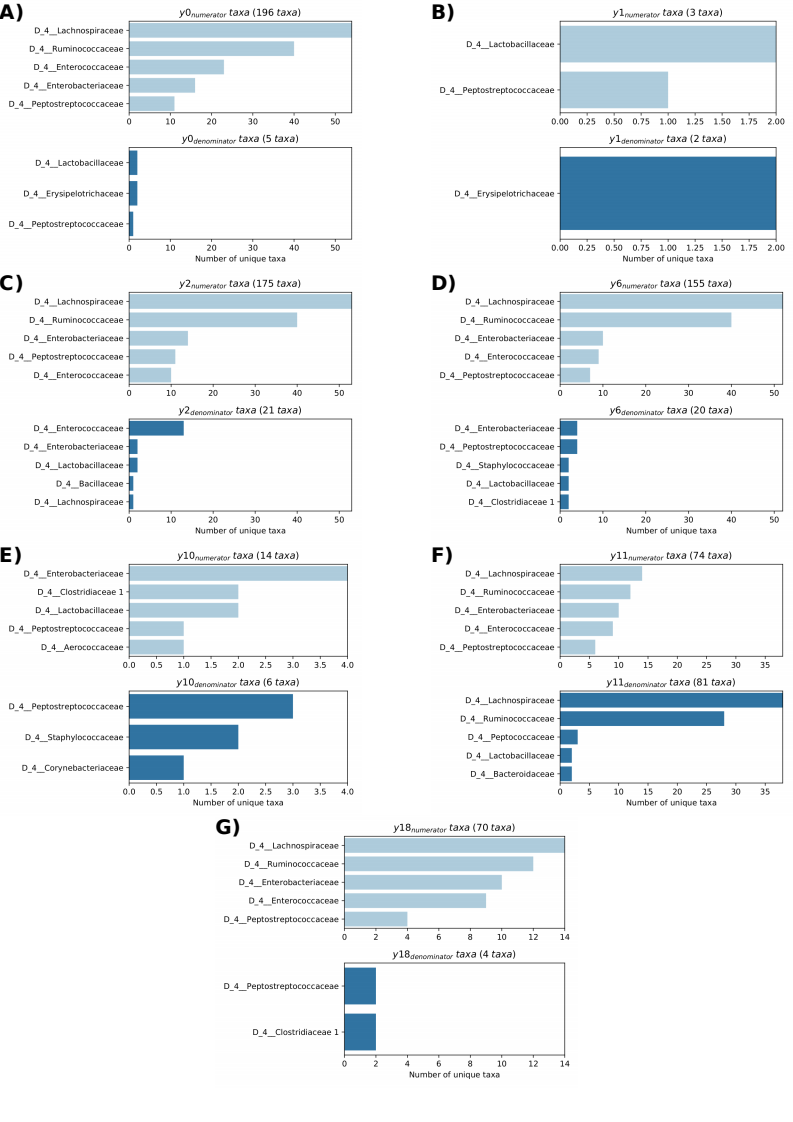


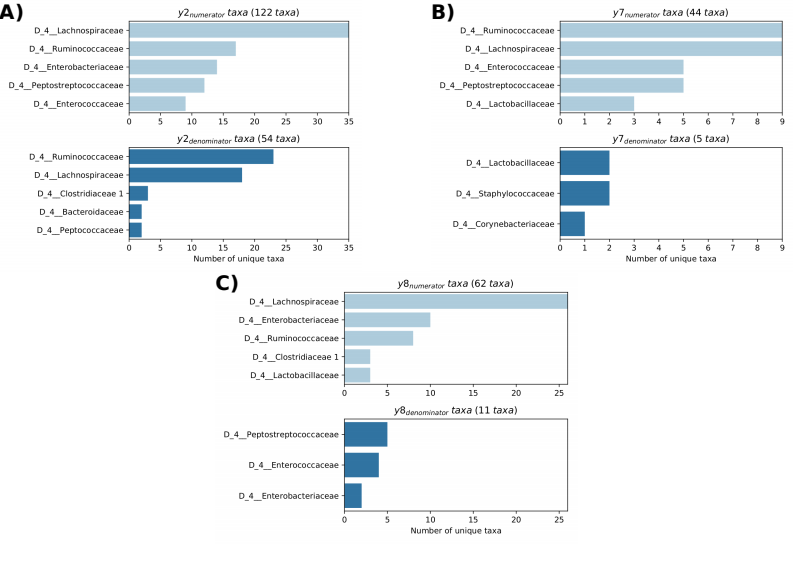
**Supplementary Figure 2.** Barplots showing the taxonomic composition of balances which reveal significant differences between lumen and mucus samples in the ileum.
